# Telehealth Hammersmith Infant Neurological Examination and early diagnosis of cerebral palsy

**DOI:** 10.1111/dmcn.70321

**Published:** 2026-05-29

**Authors:** Jennifer C. Keene, Sarah L. Winter, Tara Dupont, Lauren Ayala, Wendy G. Evans, Betsy E. Ostrander

**Affiliations:** ^1^ Department of Pediatrics, Division of Child Neurology University of Utah Salt Lake City UT USA; ^2^ Department of Pediatrics, Division of General Pediatrics University of Utah Salt Lake City UT USA; ^3^ Department of Pediatrics, Division of Neonatology University of Utah Salt Lake City UT USA

## Abstract

**Aim:**

To compare the performance characteristics of telehealth versus in‐person Hammersmith Infant Neurological Examination (HINE) assessment to support early diagnosis of cerebral palsy (CP).

**Method:**

This study is a retrospective secondary analysis of prospectively collected HINE data from a neonatal intensive care follow‐up clinic, comparing telehealth versus in‐person HINE at 3, 6, 9, and 12 months between patients with CP versus patients without CP.

**Results:**

Of 650 included children, 244 (38%) had at least one telehealth HINE and 148 (23%) were diagnosed with CP with median age at last follow‐up of 24 months. HINE score was associated with CP at 3, 6, 9, and 12 months for both telehealth and in‐person exams (telehealth odds ratio 0.74–0.90 [95% confidence interval 0.56–0.97], *p* = 0.01 to <0.001; sensitivity 50%–100% and specificity 69%–93%, in‐person odds ratio 0.74–0.86 [95% confidence interval 0.78–0.89], *p* < 0.001; sensitivity 69%–83% and specificity 88%–97%). There was no significant difference in area under the receiver operating curve between in‐person and telehealth evaluations at any age.

**Interpretation:**

Telehealth and in‐person HINE evaluation across the first year of life were both effective for supporting early CP diagnosis. Extending HINE testing to telehealth provides an opportunity to increase early diagnosis of CP in settings with limited resources and long geographic distances to specialty newborn intensive care unit follow‐up clinics.

**Abbreviations**
AUC: area under the curve
HINE: Hammersmith Infant Neurological Examination
NICU: newborn intensive care unit

**What this paper adds**

- Telehealth Hammersmith Infant Neurological Examination (HINE) scores at 3 to 12 months were associated with cerebral palsy (CP) diagnosis.
- There was no significant area under the curve difference between in‐person and telehealth HINE at 3 to 12 months.
- Telehealth HINE may improve access to early CP evaluation in resource‐limited or geographically distant settings.

Cerebral palsy (CP) is the most common motor disability in children occurring in approximately 1.6 per 1000 in high income countries.^1^, ^2^, ^3^ CP is defined as a permanent, non‐progressive disturbance in motor tone and function.^2^ Early CP diagnosis is a priority for families, facilitates access to therapeutic interventions that improve motor and cognitive development,^4^, ^5^ facilitates family/clinician communication, and improves overall quality of life for children.^6^

Standardized tools have been developed to support the identification of CP in infants with high probability of neurodevelopmental delays including the Hammersmith Infant Neurological Examination (HINE).^1^, ^7^ The HINE is a standardized and scorable neurological exam that evaluates reflexes, posture, tone, and movements, resulting in a global score^7^ and has been demonstrated to be a highly reliable tool for early CP detection and risk stratification.^1^, ^7^, ^8^, ^9^, ^10^, ^11^

Traveling to an in‐person newborn intensive care unit (NICU) follow‐up clinic can be burdensome for families, particularly in areas where there are sparse providers and for families with limited resources.^12^ The American Academy of Pediatrics supports telehealth to provide expanded access to specialty providers.^13^ Although the HINE was developed for in‐person assessment, the COVID‐19 pandemic forced a rapid pivot to telehealth for NICU follow‐up,^14^, ^15^, ^16^ with over 90% of parents satisfied with the care provided and similarly positive reviews from providers. There were similar rates of CP diagnosis in high‐risk cohorts in both telehealth‐assessed patients and matched cohorts.^16^ Sixty‐two percent of NICU follow‐up programs are planning to continue telehealth but there remain concerns about the quality of assessment, particularly neurological assessments.^12^

Although the HINE remained a component of many NICU follow‐up telehealth examinations during the pandemic, little work has been completed examining the performance of telehealth versus in‐person HINE assessments to support CP diagnosis, nor exploring the potential use of a telehealth HINE in clinics which utilize a hybrid of in‐person and telehealth NICU follow‐up. Our center serves a catchment area of approximately 400 000 square miles (the area of France and Germany combined); with a population of 1.7 million children. We have continued to offer telehealth follow‐up since the COVID‐19 pandemic, including routine telehealth HINE assessments.

This study examined the performance of telehealth versus in‐person HINE to support the early diagnosis of CP. We hypothesized that the telehealth HINE performs similarly to an in‐person HINE assessment based on our clinical experience.

## METHOD

### Study design

This study is a retrospective secondary analysis of prospectively collected data from 2018 to 2023 from all neonates with HINE assessments performed in our multispecialty neonatal follow‐up program as part of the early diagnosis of CP initiative. The University of Utah's institutional review board approved the study and consent was waived. Infants qualified for evaluation if they had any of the following: birthweight less than 1250 grams, were born at less than 27 weeks' gestation, required extracorporeal membrane oxygenation support during the neonatal period, were diagnosed with neonatal encephalopathy, had a cramped synchronized general movements assessment in the NICU,^17^ or were referred by a neurologist because of an abnormal neurological exam in the NICU. Infants were considered lost to follow‐up if their last visit with a developmental provider was at less than 12 months corrected age and they had not been diagnosed with CP.

Data on NICU stay were retrospectively extracted from chart review and included gestational age, birthweight, and if they underwent therapeutic hypothermia for neonatal encephalopathy. The grade of intraventricular hemorrhage or periventricular hemorrhagic infarct was determined by a review of clinical, radiological cranial ultrasound reports from pediatric radiologists.

Infants were assessed with a HINE at 3 months and 9 months corrected age. If there were concerns for an abnormal exam at either 3 or 9 months, then an additional exam was performed at either 6 or 12 months corrected age respectively. All exams were completed either in‐person or via telehealth with interpreters used if needed with either visit modality. Infants were evaluated by developmental pediatricians or neonatal specialized pediatric neurologists accompanied by either physical therapists or occupational therapists at each visit. The HINE consists of 26 items which are scored from 0 to 3 for a total global score of 0 to 78 as previously described.^7^ All examiners are trained to perform the HINE and undergo yearly video reviews to ensure high interobserver reliability. If an infant was seen via a virtual visit, parents were guided through the exam maneuvers with the infant fully visible on video using the assistance of a sample exam video to guide maneuvers (Video S1). Infants were included in the analysis if a HINE was missed but they had a subsequent visit establishing the presence or absence of CP. All patients are invited for 3‐ and 9‐month testing, with patients who had delayed follow‐up or who had a concerning exam at 3 months scheduled for a 6‐month visit. The 12‐month visit is also reserved for those with delayed evaluation or concerning findings at 9 months. For patients with borderline or concerning findings via telehealth at 3 or 9 months we preferentially schedule the follow‐up visit to be in‐person for a hands‐on exam.

While the optimal definition and criteria for a diagnosis of CP remains an area of active discussion in the pediatric community,^18^, ^19^ our center utilizes the guideline for early detection of CP which was developed as part of the early CP diagnosis initiative sponsored by the Cerebral Palsy Foundation. This guideline builds on the 2007 definition proposed by Rosenbaum et al.^2^ and integrates the evidence for early detection of CP as reviewed by Novak et al.^1^ Sites implemented a functional motor assessment, standardized neurological exam, and neuroimaging in high‐risk infants and demonstrated an average age at CP diagnosis of 9.5 months.^20^ HINE test results as well as clinical history and imaging were available to diagnosing clinicians. Children were assessed for the diagnosis of CP at each visit, and when the diagnosis was uncertain, patients were not diagnosed and underwent repeat examination at the next visit. Gross motor classification of impairment in patients with CP was based upon function at the latest developmental follow‐up.^21^

### Statistical analysis

Continuous variables were reported as median, first, and third quartiles and categorical variables as counts and percentages. Groups were compared using Fisher's exact and Wilcoxon rank‐sum tests. A univariate logistic regression model was used to evaluate the association between global HINE score and the odds of subsequent CP diagnosis. Multivariate logistic regression analysis was performed to adjust for demographic differences between populations who selected telehealth evaluation. Variables demonstrating a difference between in‐person and telehealth populations at a *p*‐value less than 0.1 were considered for inclusion; distance from hospital, birthweight, and diagnosis of neonatal encephalopathy were retained while gestational age was excluded from the final model because of collinearity with birthweight. Odds ratios (ORs) for HINE score are reported per 1‐point increase in the global HINE score such that OR less than 1 indicates lower odds of CP with higher HINE scores. The receiver operating curve receiver operating curve was plotted and the area under the curve (AUC) was calculated; the Youden index was used to identify the cut point maximizing combined sensitivity and specificity. For cutoff analyses, HINE scores at or below the identified threshold were classified as high risk for CP. Receiver operating curves for telehealth and in‐person evaluation were compared using the non‐parametric Delong method.^22^ The sensitivity, specificity, negative predictive value, and positive predictive value are reported for the cut point determined by the Youden index. AUC 95% confidence intervals (CIs) were calculated using 2000 stratified bootstrap replicates.^22^ Significance was defined as a *p*‐value less than 0.05. All available patient records in the cohort were reviewed; therefore, an a priori sample size was not calculated.

Analyses were performed cross‐sectionally at each age timepoint (3, 6, 9, and 12 months) relating the HINE score at each visit to eventual CP diagnosis. Infants whose last developmental follow‐up occurred before 12 months corrected age were classified as lost to follow‐up and excluded from the primary analysis. Given the relatively high proportion of infants lost to follow‐up, we conducted a sensitivity analysis to assess robustness to missing outcome data. This analysis included all infants with at least one available HINE score and assumed that infants lost to follow‐up did not subsequently receive a diagnosis of CP. This assumption was based on comprehensive access to electronic health records for all regional pediatric neurology and rehabilitation providers who diagnose and manage CP, making it unlikely that children with clinically significant CP would be missed within our health system. The sensitivity analysis was performed using the same analytic approach as the primary analysis to determine whether conclusions regarding telehealth versus in‐person HINE performance were materially altered.

Statistical analyses were conducted using RStudio (version 2024.12.0.467; RStudio, PBC, Boston, MA, USA).

## RESULTS

A total of 1070 infants had at least one HINE assessment in a neonatal follow‐up program clinic between 2018 and 2023. Four infants died between initial evaluation and 1 year of age and 419 (39%) were lost to follow‐up before 1 year corrected age (Figure S1). Infants lost to follow‐up lived slightly closer the hospital, had higher gestational age (median 30 weeks vs 29 weeks, *p* = 0.02), and birthweight (median 1200 grams vs 1100 grams, *p* = 0.03), were less likely to have intraventricular hemorrhage or periventricular hemorrhagic infarct (20% vs 28%, *p* = 0.002). Among those with intraventricular hemorrhage the grade of intraventricular hemorrhage was lower in infants lost to follow‐up (*p* < 0.001) (Table S1).

Cohort characteristics of the 650 infants included in the analysis are summarized in Table 1, comparing 245 infants with at least one HINE assessment completed via telehealth to 405 evaluated exclusively in‐person. Among the telehealth group, 102 had all exams via telehealth, 99 transitioned from telehealth to in‐person, and 44 switched to telehealth after an in‐person exam. Patients seen via telehealth lived further from the hospital (median 26 vs 22 miles, *p* = 0.02), had a lower gestational age (median 29 vs 30, *p* < 0.001), and birthweight (median 1000 grams vs 1200 grams, *p* < 0.001), and were less likely to be diagnosed with neonatal encephalopathy (19% vs 35%, *p* < 0.001). There was no difference in the frequency or distribution of diagnosis with intraventricular hemorrhage or periventricular hemorrhagic infarct. Patients diagnosed with CP were less likely to be seen by telehealth (17% vs 26% all in‐person, *p* = 0.005) although CP severity distribution was similar. Telehealth patients were significantly younger at the time of analysis (median 42 vs 61 months, *p* < 0.001). Median time at last neonatal follow‐up program follow‐up was 24 months for both groups. Table S2 describes the 148 patients diagnosed with CP including a summary of age at diagnosis, type, and severity of CP with their HINE scores over time presented in Figure S2.

**TABLE 1:** Demographic and clinical characteristics of infants undergoing telehealth versus exclusively in‐person HINE assessments.

	At least one HINE assessment via telehealth (*n* = 245)	All HINE assessments in‐person (*n =* 405)	Total (*n* = 650)	*p*
Male	128 (52)	219 (54)	347 (53)	0.69
Distance from home address to hospital (miles)^b^ Median (IQR)	26 (14–36)	22 (12–36)	23 (13–36)	0.02
Gestational age at birth (weeks) Median (IQR)	29 (26–31)	30 (27–38)	29 (26–37)	< 0.001
Birthweight (grams) Median (IQR)	1000 (820–1500)	1200 (860–2900)	1100 (850–2600)	< 0.001
Diagnosed with neonatal encephalopathy	46 (19)	142 (35)	188 (29)	< 0.001
Diagnosed with IVH or PVHI	65 (27)	120 (30)	185 (28)	0.42
Most severe intraventricular hemorrhage grade				
Grade I	25 (10)	33 (8)	58 (9)	0.32^a^
Grade II	14 (6)	24 (6)	38 (6)	
Grade III	5 (2)	17 (4)	22 (3)	
Periventricular hemorrhagic infarct	21 (9)	46 (11)	67 (10)	
Diagnosed with CP	40 (16)	108 (27)	148 (23)	0.003
GMFCS level				
I	8 (3)	22 (5)	30 (5)	0.26^a^
II	12 (5)	29 (7)	41 (6)	
III	8 (4)	23 (5)	31 (5)	
IV	11 (5)	19 (5)	30 (5)	
V	1 (0)	15 (3)	16 (2)	
Corrected age at last NFP follow‐up (months) Median (IQR)	24 (18–28)	24 (18–6)	24 (18–31)	0.04
Current age (months) Median (IQR)	42 (31–53)	61 (34–70)	48 (33–65)	< 0.001

*Note*: Data are *n* (%) unless otherwise stated.

Abbreviations: CP, cerebral palsy; GMFCS, Gross Motor Function Classification System; HINE, Hammersmith Infant Neurological Examination; IVH, intraventricular hemorrhage, IQR, interquartile range; NFP, neonatal follow‐up program; PVHI, periventricular hemorrhagic infarct.

^a^ *p*‐value calculated for overall Fisher's exact comparison between patients who did and did not ever have a HINE via telehealth.

^b^ Distance from hospital to home address missing for 17 patients ever seen by telehealth and 37 patients seen exclusively in‐person.

### Global HINE scores differentiated patients with CP both via telehealth and in‐person

The global HINE score was significantly associated with CP diagnosis at 3 months (telehealth OR 0.86 [95% CI 0.81–0.93], *p* < 0.001; in‐person OR 0.82 [95% CI 0.78–0.87], *p* < 0.001), at 6 months (telehealth OR 0.90 [95% CI 0.83–0.97], *p* = 0.01; in‐person OR 0.84 [95% CI 0.81–0.87], *p* < 0.001), at 9 months (telehealth OR 0.86 [95% CI 0.79–0.94]; *p* < 0.001; in‐person OR 0.86 [95% CI 0.81–0.87], *p* < 0.001) and at 12 months (telehealth OR 0.74 [95% CI 0.56–0.96], *p* = 0.002; in‐person OR 0.74 [95% CI 0.81–0.89], *p* < 0.001) (Table 2, Figure 1). In a multivariable model adjusting for the demographic differences between patients who opted for telehealth evaluation versus in‐person evaluation, global HINE score remained significantly associated with CP detection both via telehealth and in‐person at all timepoints except for the smallest cohort of 51 neonates evaluated by telehealth at 12 months, which was no longer significant after adjustment (Table S3). There was no significant difference in the proportion of patients with CP seen in‐person versus via telehealth at 3 or 9 months (*p* = 0.30, *p* = 0.35). At 6 months and 12 months, patients with CP were significantly disproportionately seen in‐person (*p* = 0.003, *p* = 0.008), reflecting preferential in‐person evaluation for equivocal cases.

**TABLE 2:** Comparison of telehealth and in‐person HINE scores among infants with and without subsequent cerebral palsy diagnosis.

Testing timepoint	Testing modality and scores	No CP diagnosis (*n* = 502)	CP diagnosis (*n* = 148)	Univariate odds ratio (95% CI)	*p*
3‐month testing (*n* = 267)					
	Number of telehealth (% of those tested at 3 months)	66 (34%)	19 (27%)		0.30^a^
	Number in‐person (% of those tested at 3 months)	130 (66%)	52 (73%)		
	**Telehealth global HINE score—Median (IQR)**	67 (62–69)	39 (22–48)	0.86 (0.81–0.93)	< 0.001
	**In‐person global HINE score—Median (IQR)**	56 (52–61)	40 (31–48)	0.82 (0.78–0.87)	< 0.001
6‐month testing (*n* = 397)					
	Number of telehealth (% of those tested at 6 months)	80 (26%)	10 (11%)		0.003^a^
	Number in‐person (% of those tested at 6 months)	229 (74%)	78 (89%)		
	**Telehealth global HINE score—Median (IQR)**	64 (60–68)	56 (50–63)	0.90 (0.83–0.97)	0.01
	**In‐person global HINE score—Median (IQR)**	63 (58–67)	43 (32–54)	0.84 (0.81–0.87)	< 0.001
9‐month testing (*n* = 369)					
	Number of telehealth (% of those tested at 9 months)	54 (19%)	13 (15%)		0.35^a^
	Number in‐person (% of those tested at 9 months)	226 (81%)	76 (85%)		
	**Telehealth global HINE score—Median (IQR)**	71 (64–73)	57 (43–61)	0.86 (0.79–0.94)	< 0.001
	**In‐person global HINE score—Median (IQR)**	72 (67–75)	48 (37–59)	0.86 (0.81–0.87)	< 0.001
12‐month testing (*n* = 194)					
	Number of telehealth (% of those tested at 12 months)	47 (31%)	4 (10%)		0.008^a^
	Number in‐person (% of those tested at 12 months)	107 (69%)	36 (90%)		
	**Telehealth global HINE score—Median (IQR)**	72 (68–74)	49 (43–55)	0.74 (0.56–0.96)	0.002
	**In‐person global HINE score—Median (IQR)**	72 (69–74)	50 (40–62)	0.74 (0.81–0.89)	< 0.001

*Note*: Bold indicates the scores for the two primary cohorts of interest—the Telehealth and In‐person HINE scores—in each age group, which are emphasized to highlight the primary comparison of interest.

Abbreviations: CI, confidence interval; CP, cerebral palsy; HINE, Hammersmith Infant Neurological Examination; IQR, interquartile range.

^a^ *p*‐value calculated for overall Fisher's exact comparison between patients who do and do not have CP.

**FIGURE 1:**
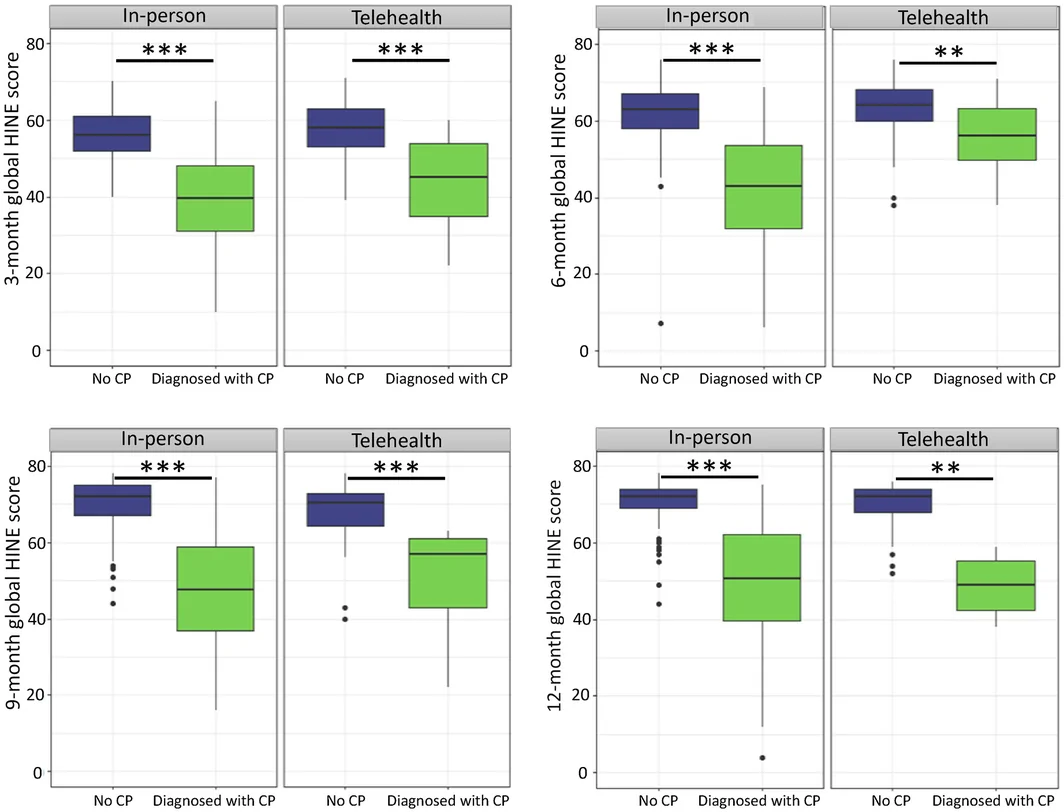
Comparison of global Hammersmith Infant Neurological Examination (HINE) scores at 3, 6, 9, and 12 months for patients with versus without cerebral palsy (CP) for visits that were conducted either in‐person or via telehealth exam. Global HINE scores were significantly different for patients with and without CP at all time timepoints and by both exam modalities. **p < 0.01. ***p < 0.001.

### Area under the receiver operating curve, sensitivity, and specificity were similar for telehealth and in‐person HINE assessments at all ages

The HINE cutoff score for predicting CP for both in‐person and telehealth exams was determined using the Youden index, selecting the threshold that maximized the combined sensitivity and specificity at 3, 6, 9, and 12 months (Figure S3). At 3 months, the in‐person cutoff was 45 (AUC 88%, specificity 97%, sensitivity 69%; positive predictive value 90%, negative predictive value 89%), whereas telehealth exams yielded a threshold of 55 (AUC 84%, specificity 69%, sensitivity 85%). At 6 months, the Youden‐index‐identified cutoff in‐person was 52 (AUC 90%, specificity 93%, sensitivity 73%) versus 54 for telehealth (AUC 74%, specificity 93%, sensitivity 50%). At 9 months, cutoffs were 63 in‐person (AUC 91%, specificity 88%, sensitivity 83%) versus 64 via telehealth (AUC 91%, specificity 78%, sensitivity 100%). At 12 months the in‐person cutoff was 63 (AUC 90%, specificity 90%, sensitivity 78%) versus 60 via telehealth (AUC 97%, specificity 92%, sensitivity 100%). There was no significant difference in the AUC at any age (Table 3) although sample size limitations may have limited our ability to detect small true differences. The sensitivity, specificity, positive predictive value, and negative predictive value in‐person and via telehealth using the global HINE cutoff values previously described to identify children at high risk for CP^10^, ^23^ as recently summarized by Fehlings et al.^23^ are included in Table S4.

**TABLE 3:** Diagnostic performance of telehealth and in‐person HINE global score cutoffs for prediction of cerebral palsy at 3‐, 6‐, 9‐, and 12‐month assessments.

Number tested at each timepoint	In‐person cutoff HINE global score	AUC (95% CI)	Specificity (95% CI)	Sensitivity (95% CI)	PPV	NPV	Telehealth cutoff HINE global score	AUC (95% CI)	Specificity (95% CI)	Sensitivity (95% CI)	PPV	NPV	*p*
3‐month (*n* = 267)	45	88% (82%–94%)	97% (81%–100%)	69% (50%–81%)	90%	89%	55	84% (75%–94%)	69% (40%–85%)	85% (53%–100%)	44%	94%	0.53^a^
6‐month (*n* = 397)	52	90% (85%–94%)	93% (80%–97%)	73% (57%–83%)	78%	91%	54	74% (56%–93%)	93% (51%–99%)	50% (10%–78%)	45%	94%	0.12^a^
9‐month (*n* = 369)	63	91% (86%–95%)	88% (66%–95%)	83% (72%–90%)	69%	94%	64	91% (84%–98%)	78% (67%–89%)	100% (54%–100%)	52%	100%	0.92^a^
12‐month (*n* = 194)	63	90% (83%–96%)	90% (58%–97%)	78% (55%–89%)	72%	92%	60	97% (93%–100%)	92% (83%–100%)	100% (25%–100%)	50%	100%	0.77^a^

Abbreviations: AUC, area under curve; CI, confidence interval; HINE, Hammersmith Infant Neurological Examination; NPV, negative predictive value; PPV, positive predictive value.

^a^ *p*‐value calculated for the comparison of AUC between telehealth and in‐person HINE assessments. Cutoff values indicated HINE scores ≤ the specified value.

### Sensitivity analysis

As described in the methods, sensitivity analyses including all infants with at least one available HINE score assuming that infants lost to follow‐up did not subsequently receive a diagnosis of CP yielded findings consistent with the primary analysis: Youden‐index‐derived cutoff scores changed by 3 points or less across timepoints and modalities compared to the primary analysis, and AUCs continued to not differ between in‐person and telehealth exams at any timepoint (Tables S5 and S6).

## DISCUSSION

In this cohort of infants with a baseline increased probability of CP, we found that a telehealth HINE and in‐person HINE assessment were both highly effective tools for supporting early diagnosis of CP and identification of infants at high probability of CP diagnosis throughout the first year of life. These findings have clinical implications and suggest we can use telehealth HINE to support families with limited local resources to identify infants who would most benefit from intensive early therapeutic intervention. Early CP diagnosis also mitigates parental and family stress and improves communication between parent and providers.^6^

We found that there were significant differences in HINE scores at 3‐, 6‐, 9‐, and 12‐month follow‐up in children with and without CP via both telehealth and in‐person evaluation (*p* = 0.01 to <0.001). This builds upon prior evidence supporting the use of in‐person HINE throughout the first year to support the detection of CP.^10^, ^24^, ^25^

The Youden‐index‐derived cutoff scores in our cohort, both by telehealth and in‐person, are similar to previously identified cutoff scores for high CP probability. Romeo et al. have previously identified that scores at 3 months corrected age of 56 or below had a sensitivity of 96% and specificity of 85%.^10^ The cutoff score via telehealth in our population was similar at 55, although we had slightly lower sensitivity (85%) and specificity (69%). Our cutoff at 6 months both in‐person and via telehealth was lower than Romeo et al. at 52 and 54 respectively with correspondingly slightly higher specificity (93% for both) and slightly lower sensitivity than Romeo et al. found using a cutoff of 59.^10^ Our cutoff scores, sensitivity, and specificity for CP identification both in‐person and via telehealth at 9 months and 12 months are also in line with the cutoff scores for high probability of CP found by Romeo et al.^10^, ^23^ Combined with the fact that there were no significant differences between the AUC between telehealth and in‐person exams at any timepoint, our findings suggest that telehealth HINE assessments performed remarkably similarly to in‐person exams throughout the first year.

There are significant pragmatic caveats to the use of telehealth HINE assessments in our cohort. At 6 months and 12 months, patients with CP were significantly more likely to be seen in‐person. This was purposeful, as patients with borderline concerning findings at the standard 3‐ and 9‐month visits were asked to travel for an in‐person subsequent visit. This highlights that despite the overall excellent performance of telehealth HINE assessments, in borderline cases an in‐person exam remains invaluable and telehealth HINE assessment is most useful when used in the setting of a clinic utilizing a hybrid of in‐person and telehealth NICU follow‐up. Furthermore, during the initial pivot to telehealth HINE assessments during the COVID‐19 pandemic our team spent a considerable amount of time working to optimize our parental coaching and explanation of the exam including creation of example videos that can be played for parents. Even with these tools and the benefit of extensive experience, obtaining a HINE score via telehealth takes more time than an in‐person evaluation. Its use is also dependent upon caregiver participation, infant cooperation, adequate physical space, reliable internet, suitable devices, and interpreter access. While these barriers may limit scalability, telehealth HINE may still extend access to early CP diagnosis to patients in resource‐limited or geographically distant settings. Recent evidence supporting HINE use in low‐ and middle‐income countries^26^, ^27^ suggests that telehealth adaptations may be promising adjuncts in this setting, although limitations in infrastructure and resources may remain barriers. Finally, a HINE is only a component of a comprehensive diagnostic evaluation for CP.

This study does have limitations. Like many studies of the HINE, this study focused on patients with a baseline high probability of neurodevelopmental delay and may not be generalizable to all NICU patients. Patients were not randomized to telehealth versus in‐person exam, and there were subtle differences in the populations who self‐assigned to each group (Table 1). This is to be expected in clinical implementation of a hybrid clinic where parental choice and characteristics will influence testing modality. While the population differences may have contributed to the subtly different performance characteristics between the two modalities, lower HINE scores were still strongly associated with CP in both modalities. We also had a relatively high loss to follow‐up with only 61% of surviving infants having follow‐up of at least a year. A sensitivity analysis incorporating all scores assuming no subsequent CP diagnosis in those lost to follow‐up showed no significant differences in outcome (Tables S4 and S5), but there is still a risk of bias as patients lost to follow‐up may not be missing at random. CP diagnosis was not blinded to prior HINE score, which may have biased the diagnosis of CP, but whether prior HINE scoring was completed by telehealth or in‐person is not readily available in the electronic health records so the method of HINE evaluation is unlikely to have significantly biased provider interpretation of prior HINE scores when making a CP determination. All children were assessed until at least 12 months of age and the median age at last follow‐up in our cohort was 24 months; however, mild CP diagnosis can occur after that time. Longitudinal evaluation may reveal future CP diagnosis among our cohort. International variability in CP classification systems may limit generalizability. Some phenotypes categorized as ‘mixed or other’ may be classified differently across regions, particularly given ongoing debate regarding hypotonia as a primary motor phenotype. Finally, in‐person and telehealth evaluation for HINE occurred with more than one trained examiner, although we attempt to standardize evaluations with yearly retraining and peer exam review.

## Conclusion

Telehealth and in‐person HINE evaluation across the first year of life were both highly effective for supporting early CP diagnosis. These results are clinically important to support the early initiation of therapies for high‐risk infants to promote optimal neurodevelopmental support in children as early as possible. Extending the use of HINE testing to telehealth provides an opportunity to increase early diagnosis of CP in settings with limited resources and long geographic distances to specialty NICU follow‐up clinics. Further work is needed to improve access to this testing to all infants with a high risk for neurodevelopmental delay to support early CP diagnosis and access to therapy.

## CONFLICT OF INTEREST STATEMENT

Sarah Winter is a consultant for ReAlta Life Sciences (Consultant). Tara Dupont is a contractor for Pfizer including contracted research.

## Supporting information


**Figure S1:** Flowchart of patient inclusion and exclusion in the study.

**Figure S2:** HINE scores at 3, 6, 9, and 12 months of age for all patients with CP.

**Figure S3:** Receiver operating curve for CP diagnosis at 3, 6, 9, and 12 months for HINE score for all patients, patients seen in‐person, patients seen via telehealth.

**Table S1:** Comparison of neonates who were or were not lost to follow‐up.

**Table S2:** CP cohort characterization.

**Table S3:** Multivariate adjusted association between HINE score and CP.

**Table S4:** Sensitivity and specificity using previously described high‐risk CP cutoff values.

**Table S5:** Sensitivity analysis including all 1066 patients assuming no CP diagnosis in those lost to follow‐up.

**Table S6:** Sensitivity analysis including all 1066 patients assuming no CP diagnosis in those lost to follow‐up.

**Video S1:** Example video of telehealth HINE assessment.

**Data S1:** STROBE statement.

## Data Availability

Deidentified individual participant data can be requested from the corresponding author subject to review by the institutional review board. The data are not publicly available because of privacy or ethical restrictions.
