# Supplementary material for: Telehealth Hammersmith Infant Neurological Examination and early diagnosis of cerebral palsy
Source: Dev Med Child Neurol. 2026 May 29;68(12):1720–8. doi: 10.1111/dmcn.70321 (PMC13743674; doi:10.1111/dmcn.70321)
Supplement: Supplementary file 1 [file DMCN-68-1720-s004.pdf]

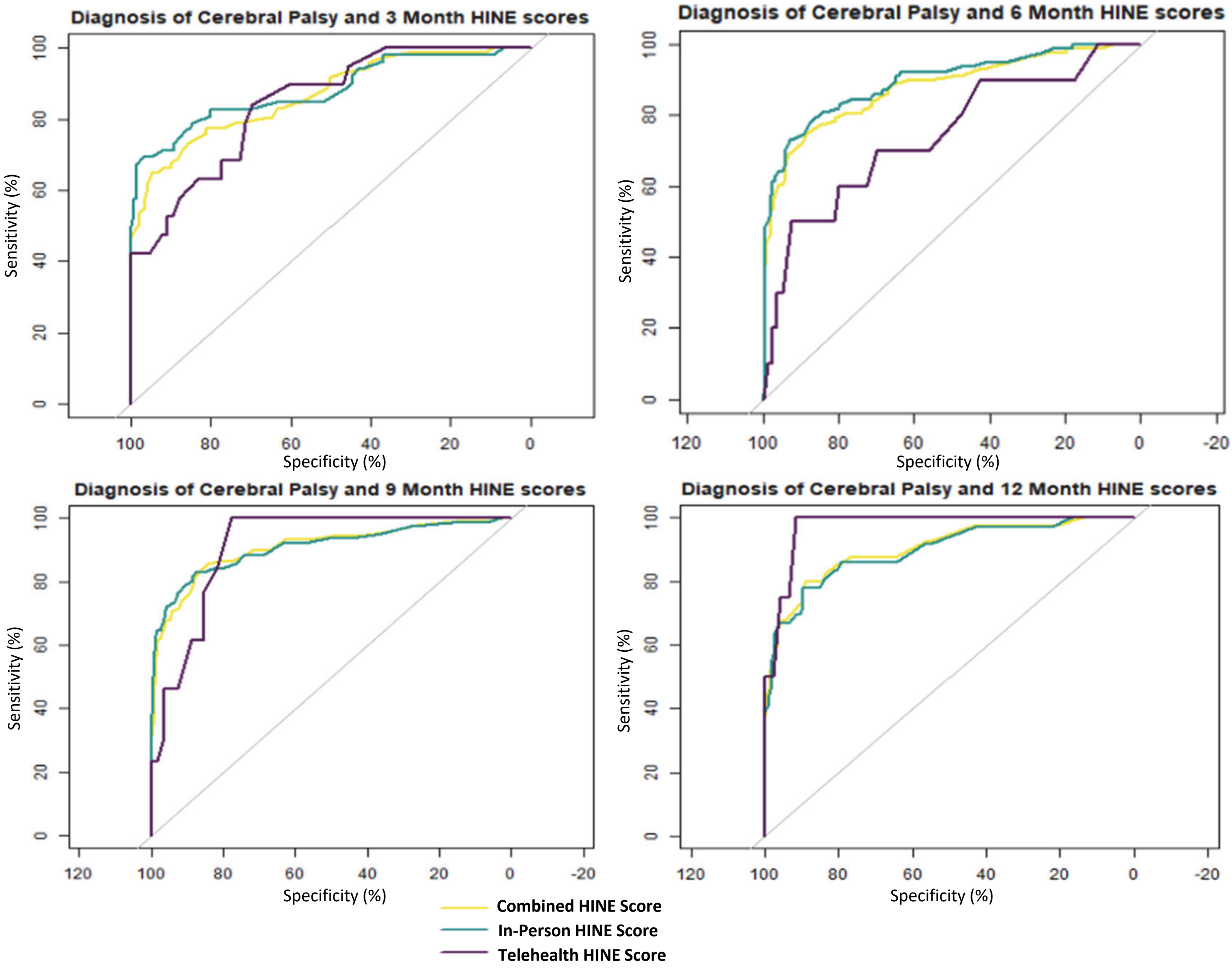
Diagnosis of Cerebral Palsy and 3 Month HINE scores
Diagnosis of Cerebral Palsy and 6 Month HINE scores
Diagnosis of Cerebral Palsy and 9 Month HINE scores
Diagnosis of Cerebral Palsy and 12 Month HINE scores
Sensitivity (%)
Specificity (%)
0
20
40
60
80
100
120
-20
Combined HINE Score
In-Person HINE Score
Telehealth HINE Score
