# Supplementary material for: Telehealth Hammersmith Infant Neurological Examination and early diagnosis of cerebral palsy
Source: Dev Med Child Neurol. 2026 May 29;68(12):1720–8. doi: 10.1111/dmcn.70321 (PMC13743674; doi:10.1111/dmcn.70321)

Figure S1 – Patient Flowchart

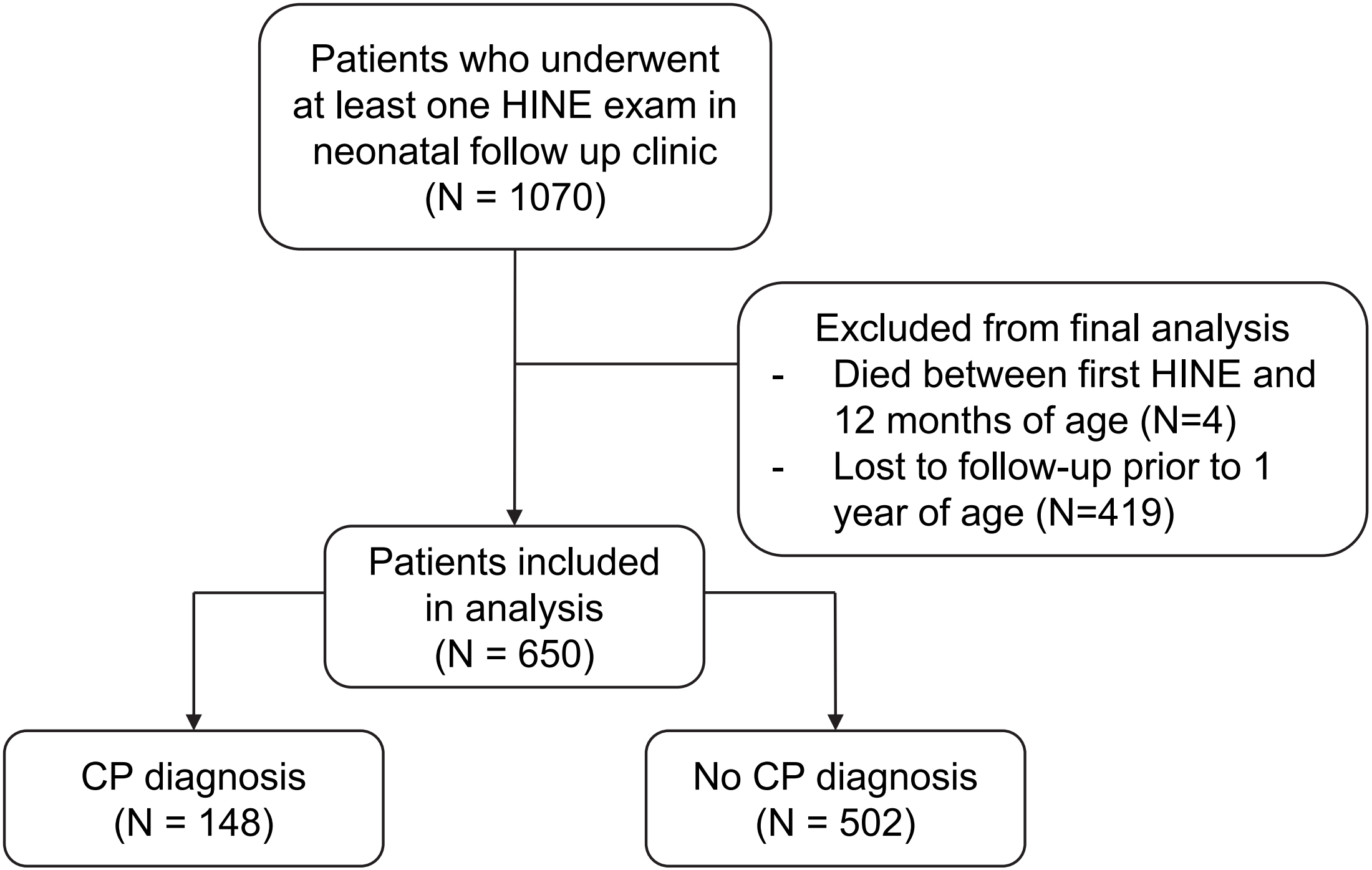

Supplement: Supplementary file 2 [file DMCN-68-1720-s008.pdf]
