# Supplementary material for: Telehealth Hammersmith Infant Neurological Examination and early diagnosis of cerebral palsy
Source: Dev Med Child Neurol. 2026 May 29;68(12):1720–8. doi: 10.1111/dmcn.70321 (PMC13743674; doi:10.1111/dmcn.70321)
Supplement: Supplementary file 3 [file DMCN-68-1720-s010.pdf]

Figure S2

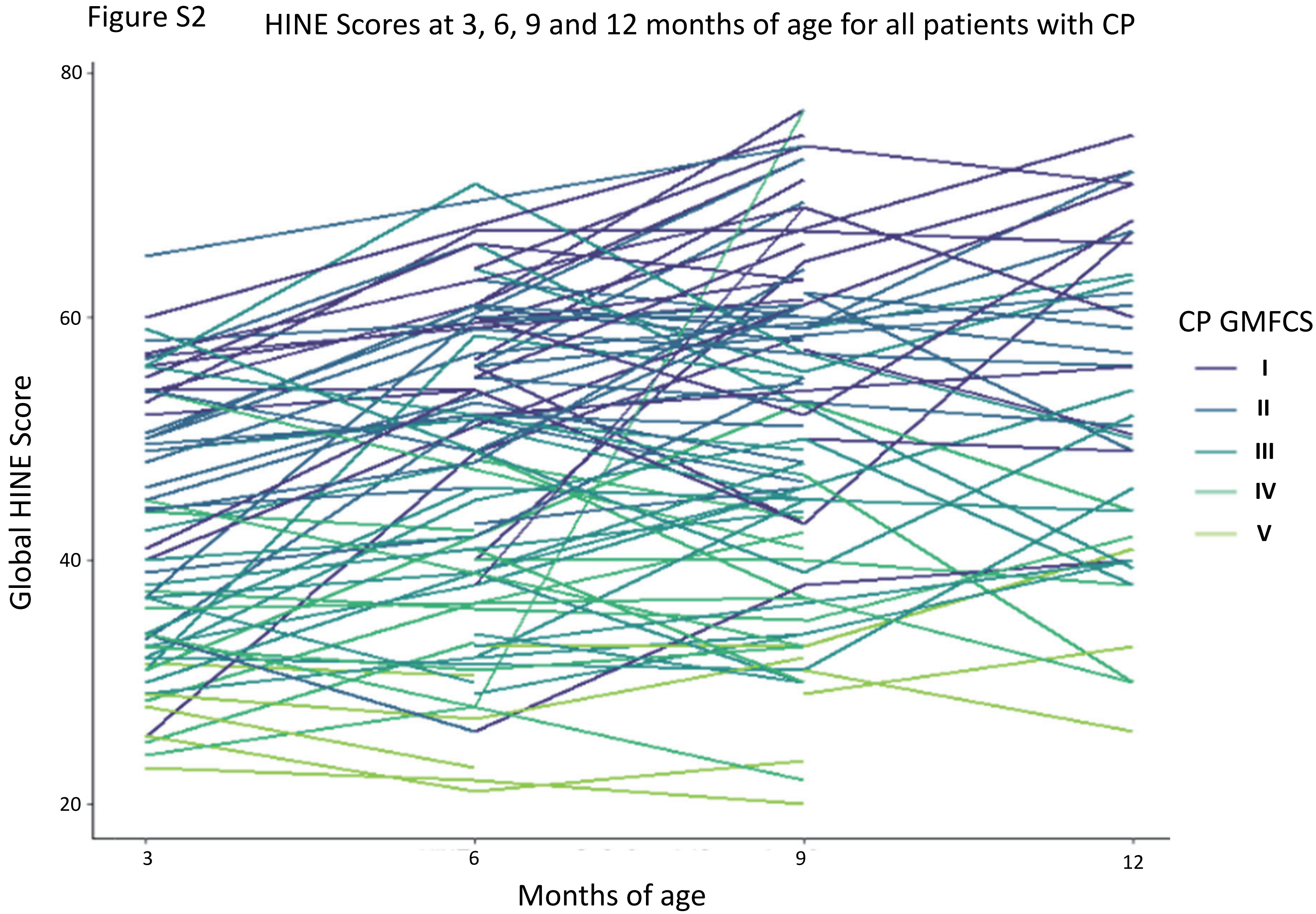

HINE Scores at 3, 6, 9 and 12 months of age for all patients with CP
Global HINE Score
80
60
40
20
3
6
9
12
Months of age
CP GMFCS
I
II
III
IV
V
