# Supplementary material for: Telehealth Hammersmith Infant Neurological Examination and early diagnosis of cerebral palsy
Source: Dev Med Child Neurol. 2026 May 29;68(12):1720–8. doi: 10.1111/dmcn.70321 (PMC13743674; doi:10.1111/dmcn.70321)
Supplement: Supplementary file 4 [file DMCN-68-1720-s001.docx]

| Table S5: Sensitivity analysis including all 1066 patients assuming no CP diagnosis in those lost to follow-up | | | | | |
| --- | --- | --- | --- | --- | --- |
|  | | |  |  |  |
|  | Testing timepoint | Testing Modality and scores | No CP diagnosis (N=918) | CP diagnosis (N=148) | P- value |
|  | **3 month testing (N = 429)** |  |  |  |  |
|  |  | **Testing Modality** |  |  |  |
|  |  | Number of Telehealth (% of those tested at 3 mo) | 147 (41%) | 19 (27%) | 0.02* |
|  |  | Number In-Person (% of those tested at 3 mo) | 211 (59%) | 52 (73%) |  |
|  |  | **Telehealth Global HINE score - Median (IQR)** | 58 (53-62) | 45 (35-54) | <0.0001 |
|  |  | **In person Global HINE score - Median (IQR)** | 56 (52-61) | 40 (31-48) | <0.0001 |
|  | **6 month testing (N = 657)** |  |  |  |  |
|  |  | **Testing Modality** |  |  |  |
|  |  | Number of Telehealth (% of those tested at 6 mo) | 199 (35%) | 10 (11%) | <0.0001* |
|  |  | Number In-Person (% of those tested at 6 mo) | 370 (65%) | 78 (89%) |  |
|  |  | **Telehealth Global HINE score - Median (IQR)** | 65 (60-69) | 56 (50-63) | 0.007 |
|  |  | **In person Global HINE score - Median (IQR)** | 63 (58-67) | 43 (32-54) | <0.0001 |
|  | **9 month testing (N = 476)** |  |  |  |  |
|  |  | **Testing Modality** |  |  |  |
|  |  | Number of Telehealth (% of those tested at 9 mo) | 92 (24%) | 13 (15%) | 0.07* |
|  |  | Number In-Person (% of those tested at 9 mo) | 295 (76%) | 76 (85%) |  |
|  |  | **Telehealth Global HINE score - Median (IQR)** | 72 (67-74) | 57 (43-61) | <0.0001 |
|  |  | **In person Global HINE score - Median (IQR)** | 72 (67-75) | 48 (37-59) | <0.0001 |
|  | **12 month testing (N = 263)** |  |  |  |  |
|  |  | **Testing Modality** |  |  |  |
|  |  | Number of Telehealth (% of those tested at 12 mo) | 71 (32%) | 4 (10%) | 0.004* |
|  |  | Number In-Person (% of those tested at 12 mo) | 152 (68%) | 36 (90%) |  |
|  |  | **Telehealth Global HINE score - Median (IQR)** | 72 (70-74) | 49 (43-55) | 0.001 |
|  |  | **In person Global HINE score - Median (IQR)** | 72 (69-75) | 51 (40-62) | <0.0001 |
