# Supplementary material for: Telehealth Hammersmith Infant Neurological Examination and early diagnosis of cerebral palsy
Source: Dev Med Child Neurol. 2026 May 29;68(12):1720–8. doi: 10.1111/dmcn.70321 (PMC13743674; doi:10.1111/dmcn.70321)
Supplement: Supplementary file 5 [file DMCN-68-1720-s002.docx]

| Table S6 - Sensitivity analysis including all 1066 patients assuming no CP diagnosis in those lost to follow-up | | | | | | |  |
| --- | --- | --- | --- | --- | --- | --- | --- |
| Number tested at each timpoint | In Person Optimally differential HINE score | AUC (95% CI) | | Telehealth Optimally differential HINE score | | AUC (95% CI) | P-value |
|  |  | |  | |  |  |  |
| 3-month  (N =429) | 45 | | 88%  (82%-94%) | | 55 | 83%  (74%-92%) | 0.45* |
|  |  | |  | |  |  |  |
| 6-month  (N = 657) | 55 | | 89%  (85%-94%) | | 54 | 75%  (58%-93%) | 0.14* |
|  |  | |  | |  |  |  |
| 9-month  (N = 476) | 63 | | 91%  (86%-95%) | | 64 | 93%  (88%-98%) | 0.53* |
|  |  | |  | |  |  |  |
| 12-month  (N = 263) | 63 | | 90%  (84%-96%) | | 60 | 98%  (95%-100%) | 0.51* |
