# Supplementary material for: Telehealth Hammersmith Infant Neurological Examination and early diagnosis of cerebral palsy
Source: Dev Med Child Neurol. 2026 May 29;68(12):1720–8. doi: 10.1111/dmcn.70321 (PMC13743674; doi:10.1111/dmcn.70321)
Supplement: Supplementary file 6 [file DMCN-68-1720-s003.docx]

Table S3 - Multivariate adjusted association between HINE score and CP

|  | Cerebral Palsy detection | | | Cerebral Palsy detection - in-person HINE | | | | Cerebral Palsy detection - telehealth HINE | | | | Cerebral Palsy detection - combined cohort | | |
| --- | --- | --- | --- | --- | --- | --- | --- | --- | --- | --- | --- | --- | --- | --- |
|  | Univariate Odds ratio | 95% CI | p-value | N | Adjusted Odds ratio* | 95% CI | p-value | N | Adjusted Odds ratio* | 95% CI | p-value | Adjusted Odds ratio* | 95% CI | p-value |
| 3 Month HINE score (N = 267) | 0.83 | 0.79-0.87 | <0.001 | 182 | 0.82 | 0.77-0.88 | <0.001 | 85 | 0.88 | 0.81-0.94 | <0.001 | 0.84 | 0.80-0.88 | <0.001 |
| 6 Month HINE score (N = 397) | 0.85 | 0.82-0.88 | <0.001 | 307 | 0.85 | 0.81-0.88 | <0.001 | 90 | 0.89 | 0.81-0.97 | 0.01 | 0.85 | 0.82-0.86 | <0.001 |
| 9 Month HINE score (N = 369) | 0.84 | 0.80-0.87 | <0.001 | 302 | 0.83 | 0.80-0.88 | <0.001 | 67 | 0.86 | 0.79-0.94 | <0.001 | 0.84 | 0.81 - 0.88 | <0.001 |
| 12 Month HINE score (N = 194) | 0.85 | 0.79-0.88 | <0.001 | 143 | 0.84 | 0.79-0.90 | <0.001 | 51 | 0.69 | 0.37-1.29 | 0.24 | 0.84 | 0.79 - 0.89 | <0.001 |

* - Logistic regression, adjusted for birthweight, presence of neonatal encephalopathy treated with therapeutic hypothermia and distance from home address to follow-up clinic in miles
