# Supplementary material for: Telehealth Hammersmith Infant Neurological Examination and early diagnosis of cerebral palsy
Source: Dev Med Child Neurol. 2026 May 29;68(12):1720–8. doi: 10.1111/dmcn.70321 (PMC13743674; doi:10.1111/dmcn.70321)
Supplement: Supplementary file 7 [file DMCN-68-1720-s005.docx]

| Table S4 - Sensitivity and Specificity using previously described high-risk CP cut-off values | | | | | | | | | | |
| --- | --- | --- | --- | --- | --- | --- | --- | --- | --- | --- |
| Number tested at each timepoint via telehealth | Previously described cut-off scores for high probability of CP | Specificity (95% CI) | Sensitivity (95% CI) | PPV | NPV | Number tested at each timepoint in-person | Specificity (95% CI) | Sensitivity (95% CI) | PPV | NPV |
|  |  |  |  |  |  |  |  |  |  |  |
| 3-month  N = 85 | 56 | 61%  (36% - 80%) | 89%  (68% - 100%) | 40% | 95% | 3-month  N = 182 | 45%  (35%-71%) | 90%  (80% - 99%) | 40% | 91% |
|  |  |  |  |  |  |  |  |  |  |  |
| 6-month  N = 90 | 59 | 60%  (43% - 93%) | 80%  (40% - 100%) | 27% | 94% | 6-month  N = 307 | 70%  (61%-87%) | 84%  (76% - 92%) | 49% | 93% |
|  |  |  |  |  |  |  |  |  |  |  |
| 9-month  N = 67 | 62 | 81%  (71% - 93%) | 85%  (46% - 100%) | 52% | 96% | 9-month  N = 302 | 78%  (59%-91%) | 89%  (79% - 93%) | 70% | 92% |
|  |  |  |  |  |  |  |  |  |  |  |
| 12-month  N = 51 | 65 | 83%  (80%-100%) | 100%  (98% - 100%) | 33% | 100% | 12-month  N = 143 | 83%  (58%-94%) | 80%  (68% - 94%) | 62% | 92% |
