# Supplementary material for: Telehealth Hammersmith Infant Neurological Examination and early diagnosis of cerebral palsy
Source: Dev Med Child Neurol. 2026 May 29;68(12):1720–8. doi: 10.1111/dmcn.70321 (PMC13743674; doi:10.1111/dmcn.70321)
Supplement: Supplementary file 8 [file DMCN-68-1720-s006.docx]

Table S2 – CP cohort characterization

|  | GMFCS I/II | GMFCS III-V | Total |
| --- | --- | --- | --- |
|  | (N=71) | (N = 77) | (N = 148) |
| **Male (%)** | 40 (56%) | 45 (58%) | 85 (57%) |
|  |  |  |  |
| **Gestational Age at birth (weeks) Median (IQR)** | 27 (25-31) | 30 (26-37) | 28 (25-35) |
| **Birth Weight (grams) Median (IQR)** | 940  (770 - 1400) | 1300  (820 - 2800) | 1100  (790 - 2300) |
|  |  |  |  |
| **Diagnosed with Neonatal Encephalopathy (%)** | 13 (18%) | 27 (35%) | 40 (27%) |
|  |  |  |  |
| **Diagnosed with IVH or PVHI (%)** | 50 (70%) | 41 (53%) | 91 (61%) |
|  |  |  |  |
| **Most severe Intraventricular Hemorrhage grade** |  |  |  |
| Grade I (%) | 5 (7%) | 7 (9%) | 12 (8%) |
| Grade II (%) | 10 (14%) | 4 (5%) | 14 (9%) |
| Grade III (%) | 5 (7%) | 8 (10%) | 13 (9%) |
| Periventricular hemorrhagic infarct (%) | 30 (42%) | 22 (29%) | 52 (35%) |
|  |  |  |  |
| **Predominant CP type** |  |  |  |
| Ataxic (%) | 2 (3%) | 0 (0%) | 2 (1%) |
| Mixed or other (%) | 6 (8%) | 10 (13%) | 16 (11%) |
| Bilateral spastic (%) | 39 (55%) | 65 (84%) | 104 (70%) |
| Unilateral Spastic (%) | 24 (34%) | 2 (3%) | 26 (18%) |
|  |  |  |  |
| **Corrected Age at time of CP diagnosis (months) Median (IQR)** | 13 (9.4-20) | 7.7 (6.4-10) | 9.5 (6.9-18) |
|  |  |  |  |
| **Corrected age at last developmental follow-up (months) Median (IQR)** | 33  (20 - 43) | 28  (19 - 50) | 28  (19 - 45) |
|  |  |  |  |
| **Corrected age at time of data analysis (months) Median (IQR)** | 47 (29 - 63) | 36 (26 - 59) | 42  (27 - 61) |
|  |  |  |  |

CP - Cerebral Palsy, GMFCS - Gross Motor Functional Classification System, IVH - Intraventricular Hemorrhage, IQR - Interquartile range (25%ile - 75%ile), PVHI = Periventricular hemorrhagic infarct
