# Supplementary material for: Telehealth Hammersmith Infant Neurological Examination and early diagnosis of cerebral palsy
Source: Dev Med Child Neurol. 2026 May 29;68(12):1720–8. doi: 10.1111/dmcn.70321 (PMC13743674; doi:10.1111/dmcn.70321)
Supplement: Supplementary file 9 [file DMCN-68-1720-s007.docx]

Table S1 – Comparison of neonates who were or were not lost to follow-up

|  | Lost to follow up before 1 year | Followed up through at least 12 months of age | Total | P-value |
| --- | --- | --- | --- | --- |
|  | (N=419) | (N = 650) | (N = 1066) |  |
| **Male (%)** | 218 (52%) | 347 (53%) | 565 (53%) | 0.8 |
|  |  |  |  |  |
| **Distance from home address to hospital (miles)  Median (IQR)**** | 23 (12-35) | 23 (13-36) | 23 (12-36) | 0.002 |
|  |  |  |  |  |
| **Gestational Age at birth (weeks) Median (IQR)** | 30 (28-35) | 29 (26-37) | 30 (27-36) | 0.02 |
|  |  |  |  |  |
| **Birth Weight (grams) Median (IQR)** | 1200  (940 - 2300) | 1100  (850 - 2600) | 1100  (880 - 2500) | 0.03 |
|  |  |  |  |  |
| **Diagnosed with Neonatal Encephalopathy (%)** | 103 (25%) | 188 (29%) | 291 (27%) | 0.14 |
|  |  |  |  |  |
| **Diagnosed with IVH or PVHI (%)** | 84 (20%) | 185 (28%) | 269 (25%) | 0.002 |
|  |  |  |  |  |
| **Most severe Intraventricular Hemorrhage grade** |  |  |  |  |
| Grade I (%) | 40 (10%) | 58 (9%) | 98 (9%) | <0.001* |
| Grade II (%) | 22 (5%) | 38 (6%) | 60 (6%) |  |
| Grade III (%) | 13 (3%) | 22 (3%) | 35 (3%) |  |
| Periventricular hemorrhagic infarct (%) | 9 (2%) | 67 (10%) | 76 (7%) |  |

IVH - Intraventricular Hemorrhage, PVHI = Periventricular hemorrhagic infarct, IQR - Interquartile range (25%ile - 75%ile), * p-value calculated for overall Fisher's exact comparison between patients who were and were not lost to follow-up, ** Distance from hospital to home address missing for 40 patients lost to follow-up and 54 patients with follow-up
